# How is dietary diversity related to haematological status of preschool children in Ghana?

**DOI:** 10.1080/16546628.2017.1333389

**Published:** 2017-06-14

**Authors:** Mahama Saaka, Sylvester Zakaria Galaa

**Affiliations:** ^a^ School of Allied Health Sciences, University for Development Studies, Tamale, Ghana; ^b^ Faculty of Integrated Development Studies, University for Development Studies, Tamale, Ghana

**Keywords:** Anaemia, minimum dietary diversity, haemoglobin, predictors of anaemia, Ghana Demographic and Health Survey

## Abstract

**Background:** The role of dietary diversity on blood biomarkers may be significant, but the evidence is limited.

**Objective:** This study assessed the association between dietary diversity and haematological status of children aged 6-59 months controlling for various known confounders.

**Design:** The analysis in this study is based on the 2014 Ghana Demographic and Health survey data.The study involved 2,388 pre-school children aged 6-59 months who constituted the sub-sample for anaemia assessment.

**Results:** The mean haemoglobin concentration (Hb) was 10.2 g/dl ± 1.50 (95 % CI: 10.1 to 10.3), and anaemia prevalence (Hb < 11 g/dl) among children aged 6-59 months was 66.8 % (CI: 63.7 to 69.8). In multivariable logistic regression analysis,continued breastfeeding [Adjusted odds ratio (AOR) = 1.9 (95% CI: 1.19–2.91], 12–23 months of age (AOR = 2.4 (95% CI: 1.40–3.98), having fever in last two weeks (AOR = 1.7 (95% CI: 1.20–2.45, birth interval ≤ 24 months (AOR = 1.9 (1.20–2.84), and poorest wealth quintile (AOR = 2.6 (95% CI: 1.48–4.48) were positively associated with anaemia.

**Conclusion:** The current study showed that factors other than poor dietary diversity predicted anaemia among children aged 6–59 months in Ghana.

## Introduction

Undernutrition, including micronutrient deficiencies, is a leading cause of maternal and child mortality and morbidity [1–5]. Of particular importance is anaemia, which remains one of the most intractable public health problems in many countries including Ghana. Anaemia is defined as a condition in which the number and size of red blood cells or haemoglobin concentration fall below an established cut-off, consequently impairing the capacity of the blood to transport oxygen around the body [6].

The global prevalence of anaemia among children under five is estimated to be about 47.4% [7]. In developing countries, it affects 46–66% of children aged under five years [8]. Anaemia has major consequences on socioeconomic development of a population [9] and it could be due to a complex interaction of many factors, including poor nutrition and health. Dietary diversity, defined as the number of different food groups consumed over a given reference period, has been identified as a useful measure for overall quality and nutrient adequacy of the diet that may influence blood formation. The dietary diversity score (DDS) is a recommended valid dietary assessment indicator that has been shown to reflect micronutrient intake among young children [10–12]. Consumption of foods from at least four food groups has been associated with a high likelihood of a child consuming at least one animal-source food and at least one fruit or vegetable, in addition to a staple food and, has been used to classify diets of children from developing countries [13,14]. Furthermore, dietary diversity is considered to be a key indicator in assessing the access, utilization and quality of diet of individuals or household [15].

In Ghana, where anaemia is of public health significance, very little is documented regarding the contribution of dietary factors to the haematological status of children. The effect of dietary diversity on blood biomarkers may be significant, but the evidence is limited. Furthermore, an association between dietary diversity and haematological status may be complicated by other factors including malarial infection and household socioeconomic status. This study therefore sought to determine the independent contribution of dietary diversity to the prevention of anaemia in children aged 6–59 months whilst controlling for potential confounding factors. We hypothesized that consumption of diversified diets would be associated with reduced prevalence of anaemia compared to nutrient-poor diets.

## Subjects and methods

### Study area

The study covered all the 10 administrative regions of Ghana. Ghana shares its northern boundary with Burkina Faso and its eastern boundary with the Republic of Togo and a western boundary with La Cote d’Ivoire.

### The study design, population and sampling

This paper is based on further analysis of data which were collected in the 2014 Ghana Demographic and Health Survey (GDHS) carried out across all 10 regions. The community-based cross-sectional survey included 2388 pre-school children aged 6–59 months who constituted the sub-sample for anaemia assessment.

Each region was considered a stratum, from which representative probability samples were selected by Demographic and Health Survey (DHS) using stratified cluster sampling methodology. The DHS sample sizes were calculated to account for separate key indicators, and clusters were selected from the master frames in the first stage via the probability proportion to size (PPS) method [16]. Households were then selected from a sampling frame using a random systematic method.

### Data collection

Study participants were then interviewed face-to-face by the investigators. Within each selected household, the caregiver responded to questions on anaemia prevention and treatment and expressed her knowledge and practices on anaemia. A pre-tested questionnaire was used to collect information including socio-demographic, infant and young child feeding (IYCF) practices, maternal knowledge, attitude, and practices on iron-rich foods, prevention and treatment of anaemia and child morbidity.

### Independent and dependent variables

The main outcome variable for this study was the prevalence of anaemia (Hb less than 11 g/dl). The independent variables were maternal, child and household characteristics, malarial infection, and child dietary intake. A brief description of main independent and dependent variables is as follows:

### Assessment of anaemia

Haemoglobin levels were determined by using a portable HemoCue 301 photometer. Trained laboratory technicians drew capillary blood samples from the finger prick with a lancet after taking all aseptic precautions. The first drop of blood was wiped away using alcohol sterile wipes, and the next drop was placed into the Hemocue curvette for immediate testing of haemoglobin. According to the World Health Association (WHO), anaemia is defined as the presence of hemoglobin level of less than 11 g/dLin children under five years of age [17]. Anaemia was further classified as mild (9.0–10.9 g/dL), moderate (7.0–8.9 g/dL) or severe (<7.0 g/dL). Anaemia is said to be a severe public health problem when its prevalence is 40% or more in any group (all types of anemia) or when severe anaemia (haemoglobin < 7 g/dL) exceeds 2% [18].

### Measurement of dietary diversity

The food groups in the DHS were regrouped to fall in line with the WHO recommended seven food groups used in defining children’s minimum dietary diversity indicator as follows: (i) grains, roots and tubers; (ii) legumes and nuts; (iii) dairy products; (iv) flesh foods (meats/fish/poultry); (v) eggs; (vi) vitamin A-rich fruits and vegetables; and (vii) other fruits and vegetables [13].

Mothers were asked to recall the number of times, in the past 24 hours, a child had received anything to eat, aside from breast milk, including meals and snacks. The dietary diversity score therefore ranged from 0–7 with minimum of 0 if none of the food groups is consumed, to 7 if all the food groups are consumed. WHO defined minimum dietary diversity as the proportion of children aged 6–23 months who received foods from at least four out of seven food groups in a 24 hour time period [13,19]. Traditionally, this concept had been applied to children 6–23 months but in this study, we extended to all children 6–59 months. We defined adequate dietary diversity as consumption of food from at least four different food groups (DDS ≥ 4).

### Socioeconomic and demographic factors

Socioeconomic and demographic information was collected on mothers’ age, marital status and highest level of education attained by the mothers. Household socioeconomic status was determined from the household wealth index. The household wealth index is a standardized asset-based score that is divided into quintiles [20]. Additional household variables included household residence (urban/rural) and household size.

### Other variables

Mothers were asked if the children were breast feeding at the time of the survey. For morbidity experience, respondents were asked to recall if the child had experienced any diarrhoea or cough episode in the past seven days preceding the interview.

### Data processing and analysis

Data were analysed using complex samples module for Windows in IBM-SPSS version 20. The analysis of data took into account the complex design of multi-stage cluster surveys. This was done in order to make statistically valid population inferences and computed standard errors from sample data. Sample weights were applied to each stratum to account for differences in population size in each (that is, weighted analysis).

Both bivariate and multivariate analyses were carried out to identify risk factors of anaemia. Association between anaemia and some risk factors in pregnancy was tested using chi-square and multivariable analysis of risk factors. Independent variables with p value less than 0.1 in bivariate analysis were entered into multivariable logistic regression model. P value less than 0.05 were taken as statistically significant and adjusted odds ratio with 95% confidence interval (CI) was used to measure association.

Analyses of association between haemoglobin concentration (Hb) and other variables were carried out using bivariate and multivariate techniques. First, bivariate analyses for all the various risk factors were performed using chi-square (χ^2^) tests for categorical variables and analysis of variance (ANOVA) for means of continuous variables.

### Ethical statement

The analyses in this paper are based on secondary data obtained with permission from MEASURE DHS Organization and was downloaded from the Demographic and Health Surveys (DHS) online archive. DHS datasets are in the public domain and available to all registered users who have been granted access upon request. The original DHS data were collected with approval from the Inner City Fund (ICF) International’s Institutional Review Board and national ethical guidelines. Information about objective of the study, procedures, potential risks and benefits was given to mothers before their children were enrolled to the study. Verbal informed consent was obtained before the household questionnaires were administered, and before blood was collected for anemia testing. An informed consent was read in the local language and a copy given to the household upon request. Those selected to give blood samples were informed of the general purpose, possible risks and benefits of the survey in their language. Participation in the survey was voluntary and participants’ full right to refuse participation was explained.

## Results

### Sociodemographic characteristics of the sample

A total of 2388 children aged 6–59 months were involved in this analysis; 52.9% were male, and 47.1% were female. The mean age of the children was 31.5 ± 15.3 months. Majority of the respondents (51.3%) had at least secondary education and 20.5% fall within the poorest household wealth index. Petty trading and agricultural farming were the common occupations among the mothers. Table 1 displays the socio-demographic characteristics of the study sample.

**Table 1.** Sociodemographic characteristics of sample (N = 2388).

Characteristics	Frequency (n)	Percentage (%)
**Age of children (months)**		
6–11	268	11.3
12–23	580	24.5
24–35	550	22.5
36–47	504	21.5
48–59	486	20.2
**Gender of child**		
Male	1246	52.9
Female	1142	47.1
**Educational level of mothers**		
No education	865	28.9
Primary	490	19.7
Secondary	960	48.1
Higher	73	3.2
**Household wealth index**		
Poorest	774	22.8
Poorer	516	20.5
Middle	448	20.0
Richer	354	18.3
Richest	296	18.4
**Occupation of respondent**		
Not working	381	16.8
Professional/technical/managerial	86	4.0
Clerical	13	0.6
Sales	761	36.2
Agricultural – self-employed	787	27.4
Agricultural – employee	22	0.7
Services	19	0.8
Skilled manual	290	12.2
Unskilled manual	28	1.4

### Prevalence of anaemia

The mean haemoglobin concentration among the children studied was 10.2 g/dl ± 1.50 (95% CI: 10.1–10.3), and an overall prevalence of anaemia (Haemoglobin level < 11 g/dl) among children aged 6–59 months was 66.8% (CI: 63.7–69.8). In terms of severity, mild anaemia was 27.0% (95% CI: 24.9–29.2), moderate anaemia was 37.4% (CI: 34.6–40.3) and severe anaemia was 2.4% (CI: 1.8–3.3).

The prevalence of anaemia decreased with age and the highest level was in the Northern Region.

Overall, there was no gender difference for anaemia in the study sample. The proportion of children suffering from anaemia according to age group, gender and geographical location is shown in Table 2.

**Table 2.** Prevalence of anaemia by age, gender and geographical location.

Age (months)	Total no.	Mean Hb (g/dL)	Total anaemia (Hb < 11.0 g/dL) n (%)	Severe (< 7.0 g/dL). n (%)	Moderate (7.0–8.9 g/dL) n (%)	Mild (9.0–10.9 g/dL), n (%)
6–11	268	9.8 ± 1.5	209 (78.4)	12 (3.8)	129 (48.3)	68 (26.3)
12–23	580	9.7 ± 1.5	460 (76.9)	27 (4.3)	284 (47.9)	149 (24.7)
24–35	550	10.3 ± 1.5	386 (66.0)	12 (1.5)	212 (33.7)	162 (30.7)
36–47	504	10.5 ± 1.5	323 (61.6)	10 (1.8)	168 (32.0)	145 (27.4)
48–59	486	10.7 ± 1.4	284 (55.0)	6 (1.1)	148 (28.4)	130 (25.5)
**Gender**						
Male	1246	10.2 ± 1.5	886 (66.7)	44 (2.7)	494 (36.6)	348 (27.5)
Female	1142	10.2 ± 1.5	776 (66.9)	23 (2.1)	447 (38.3)	306 (26.4)
**Region**						
Western	247	10.2 ± 1.6	168 (67.7)	10 (3.3)	85 (34.6)	73 (29.8)
Central	253	9.9 ± 1.5	184 (73.4)	8 (2.2)	107 (44.5)	69 (26.7)
Greater Accra	185	10.6 ± 1.4	111 (58.4)	2 (1.3)	53 (28.3)	56 (28.8)
Volta	187	10.1 ± 1.5	131 (70.7)	4 (2.1)	75 (41.3)	52 (27.3)
Eastern	206	10.3 ± 1.4	140 (66.5)	2 (0.9)	77 (37.6)	61 (28.0)
Ashanti	234	10.7 ± 1.6	123 (54.1)	5 (2.0)	59 (27.3)	59 (24.8)
Brong Ahafo	284	10.4 ± 1.6	183 (63.5)	8 (2.6)	92 (31.4)	83 (29.5)
Northern	377	9.4 ± 1.4	320 (84.5)	14 (3.7)	217 (57.8)	89 (22.9)
Upper East	218	10.0 ± 1.4	159 (74.6)	5 (2.9)	89 (41.0)	65 (30.6)
Upper West	197	9.8 ± 1.6	143 (73.6)	9 (7.5)	87 (44.8)	47 (21.4)

### Relationship between minimum dietary diversity and anaemia

The relationship between dietary diversity and anaemia was investigated among children aged 6–59 months. The mean dietary diversity score (DDS) from seven food groups was 2.0 ± 1.5 (95% CI: 1.9–2.1). Analysis of covariance (ANCOVA) showed that a very strong interaction existed between minimum dietary diversity and age group on mean Hb, F (2, 1373) = 18.69, P < 0.001. This meant that the association between dietary diversity and Hb varied according to age group and the interaction was greatest in the age group 48–59 months. Minimum dietary diversity was positively associated with mean Hb concentrations among children 6–23 months but it was negatively associated with Hb concentrations among children 48–59 months (Table 3a). Minimum dietary diversity was weakly associated with anaemia prevalence only among children 6–23 months (Table 3b).

**Table 3.** (a) Relationship between minimum dietary diversity and mean Hb stratified by age of child. (b) Relationship between minimum dietary diversity and prevalence of anaemia stratified by age of child.

(a)					
	Age in months				
	6–11	12–23	24–35	36–47	48–59
	268	579	105	197	224
N	Mean (95% CI)	Mean (95% CI)	Mean (95% CI)	Mean (95% CI)	Mean (95% CI)
**Variable**					
Minimum diet diversity					
< 4	9.7 (9.48, 9.87)	9.6 (9.46, 9.74)	10.1 (9.86, 10.42)	10.1 (9.86, 10.34)	10.5 (10.27, 10.66)
≥ 4	10.3 (9.80, 10.70)	9.9 (9.64, 10.13)	9.8 (8.01, 11.62)	10.2 (9.58, 10.72)	9.8 (9.35, 10.32)
**Test Statistic**	F (1, 267) = 4.0, p = 0.047	F (1, 578) = 3.8, p = 0.05	F (1, 104) = 0.3, p = 0.6	F (1, 196) = 0.03, p = 0.9	F (1, 223) = 6.3, p = 0.01

(b)				
		6–11 months		
		Anaemia		
Characteristic	N	No n (%)	Yes n (%)	Test statistic
**Minimum diet diversity**				
< 4	238	50 (21)	188 (79.0)	χ^2^ = 1.3; p = 0.3
≥ 4	30	9 (30.0)	21 (70.0)	
		**12–23 months**		
**Minimum diet diversity**				
< 4	426	80 (18.8)	346 (81.2)	χ^2^ = 3.7; p = 0.05
≥ 4	153	40 (26.1)	113 (73.9)	
		**24–35 months**		
**Minimum diet diversity**				
< 4	98	32 (32.7)	66 (67.3)	χ^2^ = 0.1; p = 0.8
≥ 4	7	2 (28.6)	5 (71.4)	
		**36–47 months**		
**Minimum diet diversity**				
< 4	168	50 (29.8)	118 (70.2)	χ^2^ = 0.3; p = 0.6
≥ 4	29	10 (34.5)	19 (65.5)	
		**48–59 months**		
**Minimum diet diversity**				
< 4	190	65 (34.2)	125 (65.8)	χ^2^ = 0.3; p = 0.6
≥ 4	34	10 (29.4)	24 (70.6)	

### Analysis of haemoglobin levels based on the type of food groups consumption frequency among children of 6–59 months

Analysis of haemoglobin levels based on the type of foods fed to the children aged 6–59 months is shown in Table 4. Continued breastfeeding was directly associated with reduced haemoglobin concentration. Consumption of staple food (cereals, roots and tubers) and vitamin A-rich fruits and vegetables in the past 24 hours was associated with reduced haemoglobin concentrations. Consumption of the other food groups in the past 24 hours prior to the study was not associated with haemoglobin concentration levels.

**Table 4.** Association between dietary factors and mean Hb among children 6–59 months.

				95% Confidence interval for mean		
Indicator	N	Mean Hb	Std deviation	Lower bound	Upper bound	Test Statistic
**Is child currently breastfeeding?**						
Yes	1290	9.8	1.5	9.76	9.92	F (1, 2387) = 89.6, p < 0.001
No	1098	10.4	1.5	10.34	10.52	
**Consumption of staple food (cereals, roots and tubers)**						
No	371	10.1	1.5	9.96	10.27	F (1, 1372) = 10.63, p = 0.001
Yes	1002	9.8	1.5	9.72	9.91	
**Consumption of vitamin A-rich fruits and vegetables**						
No	852	10.0	1.5	9.87	10.07	F (1, 1372) = 5.47, p = 0.02
Yes	521	9.8	1.5	9.64	9.90	
**Other vegetables**						
No	1159	9.9	1.5	9.80	9.98	F (1, 1372) = 0.24, p = 0.6
Yes	214	9.9	1.5	9.74	10.14	
**Flesh meat (e.g. red meats, fish, etc.)**						
No	775	9.9	1.6	9.83	10.05	F (1, 1372) = 1.4, p = 0.2
Yes	598	9.8	1.5	9.72	9.96	
**Eggs**						
No	1170	9.9	1.5	9.79	9.96	F (1, 1372) = 1.4, p = 0.2
Yes	203	10.0	1.4	9.81	10.21	
**Legumes**						
No	1192	9.9	1.5	9.80	9.97	F (1, 1372) = 0.7, p = 0.4
Yes	181	10.0	1.6	9.76	10.21	
**Dairy products**						
No	1357	9.9	1.5	9.82	9.98	F (1, 1372) = 2.0, p = 0.2
Yes	16	9.4	1.7	8.42	10.29	

### Factors associated with anaemia

Bivariate analyses were performed to assess association of sociodemographic and other factors with child anemia (Table 5). The overall prevalence of anaemia fell with age and was significantly associated with religion of the mother and whether or not the child was breastfeeding at the time of the survey. A smaller proportion of still-breastfeeding than non-breastfeeding children consumed the recommended minimum dietary diversity (16.5 versus 44.0%) (χ^2^ = 73.7; P < 0.001).

**Table 5.** Bivariate analysis of predictors of anaemia among children aged 6–59 months.

		Anaemia		
Characteristic	N	Non (%)	Yesn (%)	Test statistic
**Is child currently breastfeeding?**				
No	1098	412 (41.1)	686 (58.9)	χ^2^ = 57.6; p < 0.001
Yes	1290	314 (26.4)	976 (73.6)	
**Is child currently stunted?**				
No	1875	623 (36.2)	1252 (63.8)	χ^2^ = 40.3; p < 0.001
Yes	497	99 (20.7)	398 (79.3)	
**Age (months)**				
6–11	268	59 (21.6)	209 (78.4)	χ^2^ = 81.3; p < 0.001
12–23	580	120 (23.1)	460 (76.9)	
24–35	550	164 (34.0)	386 (66.0)	
36–47	504	181 (38.8)	323 (61.2)	
48–59	486	202 (45.0)	284 (55.0)	
**Maternal education**				
None	865	175 (19.9)	690 (80.1)	χ^2^ = 100.1; p < 0.001
Primary	490	145 (31.7)	345 (68.3)	
Secondary	960	366 (40.3)	594 (59.7)	
Higher	73	40 (56.2)	33 (43.8)	
**Household wealth index**				
Poorest	774	156 (19.2)	618 (80.8)	
Poorer	516	127 (23.4)	389 (76.6)	χ^2^ = 143.6; p < 0.001
Middle	448	155 (36.7)	293 (63.3)	
Richer	354	131 (39.9)	223 (60.1)	
Richest	296	157 (51.0)	139 (49.0)	
**Religion**				
Christianity	1676	577 (36.6)	1099 (63.4)	χ^2^ = 42.0; p < 0.001
Islam	519	118 (26.0)	401 (74.0)	
African traditional religion	193	31 (15.7)	162 (84.3)	
**Type of place of residence**				
Urban	958	357 (40.5)	601 (59.5)	χ^2^ = 48.9; p < 0.001
Rural	1430	369 (27.0)	1061 (73.0)	
**Place of delivery**				
Home delivery	749	148 (21.4)	601 (78.6)	χ^2^ = 54.8; p < 0.001
Institutional delivery	1639	578 (37.5)	1061 (62.5)	
**Classification of ANC visits**				
< 4	218	37 (17.3)	181 (82.7)	χ^2^ = 22.2; p = 0.001
≥ 4	1491	466 (34.0)	1025 (66.0)	
**Anaemia status of mother**				
No	1329	478 (38.1)	851 (61.9)	
Yes	1041	242 (26.7)	799 (73.3)	χ^2^ = 34.6; p < 0.001
**Parity of mother**				
1–2	833	281 (37.6)	552 (62.4)	χ^2^ = 16.1; p = 0.008
3–4	815	251 (33.0)	564 (67.0)	
> 4	740	194 (28.1)	546 (71.9)	
**Diarrhoea in the past two weeks**				
No	2072	657 (34.1)	1415 (65.9)	χ^2^ = 6.3, p = 0.07
Yes	316	69 (26.8)	247 (73.2)	
**Had fever in last two weeks**				
No	1992	643 (35.4)	1349 (64.6)	χ^2^ = 28.8; p < 0.001
Yes	396	83 (21.0)	313 (79.0)	

Christians had a lower prevalence of anaemia than moslems and traditionists. Both educational level and household wealth quintiles were negatively associated with anaemia.

### Multivariable analysis of the determinants of anaemia among children 6–59 months

In bivariate analyses, stunting and minimum dietary diversity were associated with anaemia but they did not remain in a regression model. Multivariate logistic regression analysis showed that continued breastfeeding (AOR = 1.9 (95% CI: 1.19–2.91), 24–35 months of age (AOR = 2.5 (95% CI: 1.64–3.83), 12–23 months of age (AOR = 2.4 (95% CI: 1.40–3.98), having fever in last two weeks (AOR = 1.7 (95% CI: 1.20–2.45), anaemic mother (AOR = 1.6 (95% CI: 1.21–2.04), birth interval ≤ 24 months (AOR = 1.9 (1.20–2.84), Home delivery (AOR = 1.4 (95% CI: 1.04–2.00), ANC attendance << 4 (AOR = 1.5 (95% CI: 1.01–2.39), no toilet facility (AOR = 1.5 (95% CI: 1.05–2.11) and poorest wealth quintile (AOR = 2.6 (95% CI: 1.48–4.48) were positively associated with anemia (Table 6). The set of variables could explain only 17% of the variation in anaemia in the population (Nagelkerke R square = 0.170) which suggest many more factors other than those measured in this study are responsible for anaemia.

**Table 6.** Predictors of anaemia among children stratified by age groups (multivariable analysis).

Age group (months)	6–23	24–59	6–59
Variable	AOR (95% CI)	AOR (95% CI)	AOR(95% CI)
Minimum			
**≥4**	Reference	Reference	Reference
**<4**	Not significant in final model	Not significant in final model	Not significant in final model
**Household wealth index**			
Poorest quintile	1.7 (0.71, 4.26)	3.0 (1.48, 6.05)**	2.6 (1.48, 4.48)***
Poorer	2.0 (0.84, 4.57)	2.5 (1.29, 4.81)**	2.4 (1.43, 4.03)***
Middle	1.2 (0.51, 2.76)	2.1 (1.10, 3.95)*	1.9 (1.15, 3.20)**
Richer	2.2 (0.99, 4.96)	2.6 (1.42, 4.90)**	2.7 (1.66, 4.46)***
Richest	Reference	Reference	Reference
**Is mother anaemic?**			
No	Reference	Reference	Reference
Yes	1.6 (1.07, 2.50)*	1.5 (1.05, 2.02)*	1.6 (1.21, 2.04)***
**Currently breastfeeding?**			
No	Reference	Reference	Reference
Yes	1.4 (0.80, 2.41)	3.0 (1.38, 6.54)**	1.9 (1.19, 2.91)***
**Type of toilet facility**			
			
No toilet facility	1.8 (1.02, 3.18)*	1.4 (0.89, 2.14)	1.5 (1.05, 2.11)*
Flush toilet	0.8 (0.38, 1.63)	1.2 (0.67, 2.06)	1.0 (0.63, 1.53)
Pit latrine	Reference	Reference	Reference
**Fever in the past two weeks?**			
No	Reference	Reference	Reference
Yes	2.9 (1.45, 5.95)**	1.40 (0.92, 2.12)	1.7 (1.20, 2.45)**
**Frequency of ANC visits**			
0–3	1.7 (0.80, 3.73)	1.41 (0.82, 2.43)	1.5 (1.01, 2.39)*
At least 4	Reference	Reference	Reference
**Place of delivery**			
Home delivery	1.9 (1.08, 3.26)*	1.2 (0.77, 1.73)	1.4 (1.04, 2.0)*
Institutional delivery	Reference	Reference	Reference
**Birth interval in months**			
> 24	Reference	Reference	Reference
Up to 24	2.5 (1.17, 5.39)**	1.7 (1.02, 2.93)*	1.9 (1.20, 2.84)**

*significant at p < 0.05; **significant at p < 0.01; ***significant at p < 0.001.

AOR (95% CI): Adjusted odds ratio at 95% confidence level.

## Discussion

The study is the first to investigate the potential role dietary diversity plays in haematological status of children using a nationally representative sample in Ghana. The analyses sought to assess the association between dietary diversity to haematological status of children aged 6–59 months whilst controlling for potential confounding factors including malarial infection.

The most consistent determinants of anaemia were child’s age, continued breastfeeding, poverty, having fever, anaemic mother, birth interval ≤ 24 months, home delivery and having no access to toilet facility. Genetic characteristics and micronutrient interactions may impair normal haemoglobin synthesis but these were not assessed in this survey.

### Prevalence of anaemia

The prevalence of anaemia among children aged 6–59 months was 66.8%. Anaemia is a public health problem when more than 5% of the population is anaemic, a significant public health problem in need of immediate action when prevalence exceeds 20%, and a severe public health problem when prevalence exceeds 40% [18]. The survey findings corroborate past research that in developing countries, anaemia affects 46–66% of children aged under five years [18].

Most of the children in this study had anaemia of mild to moderate severity with very few being severely anaemic. According to the WHO, the most common cause of high prevalence of anemia (above 40%) is the lack of dietary iron, which is related to the low consumption of this micronutrient and/or to the high ingestion of inhibitors of iron absorption [21]. Iron deficiency is the most common preventable nutritional deficiency in the world, especially among infants and young children in developing countries [22] and yet it is one of the most intractable public health problems in most countries.

### Relationship between dietary diversity and anaemia

Evidence from the analyses of the data showed that there was an interaction between minimum dietary diversity and age group on mean Hb. This meant the association between dietary diversity and Hb varied according to age group. In bivariate analyses, minimum dietary diversity was positively associated with mean Hb concentrations among children 6–23 months but it was negatively associated with Hb concentrations among children 48–59 months. Minimum dietary diversity was weakly associated with reduced prevalence of anaemia only among children 6–23 months although this relationship disappeared in the multiple regression model. This apparent lack of association may be due to the fact that the consumption of iron-rich foods was generally low in the study sample. Consequently, statistical power to detect significant associations between anaemia and dietary diversity was low. It should be noted, however, that diet is only one of the protective factors against anaemia and dietary diversity may not be the most pressing constraint in the Ghanaian population where infections are also common.

The relationship between dietary diversity and anaemia has been inconclusive. Although intake of diversified foods is expected to protect against anaemia, some other studies have reported a lack of association between dietary diversity and anaemia, especially in environments where many other factors other than diet exposure can increase the risk of getting anaemia. Neither minimum dietary diversity as a composite index, nor intake of specific food groups (e.g. grains, vegetables, flesh food and eggs) is reported to be unrelated to anaemia in several studies [23–26].

Dietary diversity at the individual level is a measure of quality of the diet. That is, the more diversified a child’s diet is, the larger the variety of nutrients he/she receives which enhance his/her health and nutrition. The number of different food groups consumed therefore better reflects a quality diet. Children who consume, for example, an average of four different food groups implies that their diets offer some diversity in both macro- and micronutrients intakes.

It must be noted that, although no strong associations were found between anaemia and dietary diversity in the present study, at least one study has shown significant association between dietary diversity and anaemia in preschool children [27].

We also noted in our study that household wealth index associated positively with minimum dietary diversity (MDD) (chi-square = 21.2, p < 0.001) and once household wealth index is in the model, the effect of MDD becomes insignificant. It is possible wealth index could be replacing the role of MDD and so studies that have not controlled for wealth index could conclude that MDD is negatively associated with anaemia.

## Dietary intake of children and prevalence of anaemia among children of 6–59 months

Breast milk generally is low in iron, since iron store in breast milk can only sustain the child from birth to 6 months [14]. This means that children who do not receive adequate complementary foods rich in iron stand a greater risk of anaemia. Micronutrient deficiency is considered one of the main causes of anaemia [28]. Iron deficiency is by far the major cause of nutritional anaemia worldwide [28].

Continued breastfeeding was directly associated with poor dietary diversity score and reduced haemoglobin concentration. Several studies have earlier reported of the negative association between continued breastfeeding and the risk of anaemia and poor complementary feeding [11,29–32].

Increased dietary diversity is associated with a higher likelihood of meeting children’s recommended nutrient intake levels [10] that may include important nutrients such as iron and zinc. It is not surprising that continued breastfeeding children were more likely to be anaemic because of the associated poor diets. The negative relationship between breast feeding and adequate DDS has been reported in other countries [33,34] and has policy implications for IYCF promotion efforts. Breastfeeding women should be specially targeted for improved dietary practices. Breastfeeding mothers may have the erroneous impression that their children are having adequate dietary intake. It is possible that such mothers were not aware of the importance of adequate complementary foods in meeting young children’s nutritional needs. It is well documented that although breast milk contributes significantly to the total nutrient intake of children within 6–24 months of age, it may not be an adequate source of micronutrients such as iron, zinc and vitamin A for older children, especially in the presence of maternal deficiency [6,35].

Cereals, roots and tubers were the predominant foods consumed and these types of food contain a high content of iron inhibitors such as polyphenols and provide low amounts of bioavailable iron [36,37]. This perhaps explains the negative association between consumption of these foods and Hb concentrations observed in this study.

Nutritional problems, including anaemia, are common in poor populations, since their diets are predominantly based on starchy staples [38] and these plant-based diets are low in micronutrient contents, and high in phytate and dietary fibre which inhibit the absorption of micronutrients such as iron [39].

From the analysis we found that children who were breastfeeding were more likely to be anaemic as compared to their counterparts who were not breastfeeding. This finding may be explained by the fact that, breast milk alone is inadequate to meet the nutritional needs of children aged 6–24 months but rather a combination of breastfeeding and adequate complementary foods is needed [40].

According to the results of the current survey, it appears that breastfeeding children aged 6–23 months were not getting adequate complementary foods. In the study sample, the dietary diversity score of such children was significantly lower than their counterparts who were not breastfeeding. All these point to the fact that breastfeeding children are at greater risk of being fed on sub-optimal complementary feeding regime and this has great potential of compromising their iron status.

### Determinants of anaemia among children 6–59 months

In the study, young children were more likely to be anemic compared with older children. The overall prevalence of anaemia was inversely related to age and the greatest prevalence occurred among the 6–11 months age group. This finding is consistent with the literature which maintains that anaemia is common among children around the time of the growth spurt, especially between the ages of 6 and 24 months [36,41–43]. The child’s physical development is rapid during this period, thereby requiring an expanded blood volume. At this stage of growth, if the iron storage from the maternal source is depleted then diet becomes a vital source for iron as a result [28]. This means that if exogenous iron is deficient, anaemia can occur easily [23].

Several other studies in many countries including India and Burma have reported a similar effect of the age of the child on anaemia [31,32,44]. Younger children are most vulnerable to anaemia perhaps because they require a relatively higher iron intake to meet the demand of rapid growth.

In addition, most complementary foods do not supply enough iron for children aged 6–11 months [45]. So, iron-rich complementary foods need to be introduced to children at six months, when maternal iron stores are exhausted and after exclusive breastfeeding [45]. Additionally, some infectious diseases including malaria and diarrhoea may have greater effect on such children, so preventing and treating these infections timely is also important for childhood anemia in this age.

Evidence from our analysis showed children from families of poorest wealth quintiles were more likely to be anaemic than children of the richest wealth quintile. The finding is in line with studies conducted in other countries including Brazil [32] and Bangladesh [27,46]. Poor household economic status might constrain families to access available health services such as antenatal care, decent toilet facilities and the power to purchase diversified and nutrient rich foods, all of which are risk factors to anaemia.

Stunted children were more likely to be anaemic than their counterparts. This finding is similar to studies conducted in many countries including Ethiopia, Bangladesh, Brazil and Burma [3,4,23,27]. The reverse equally holds whereby anaemic children are more likely to be undernourished [4] because a low hemoglobin level negatively affects linear growth [47].

Consistent with other studies [23,46], maternal anaemia was associated with anemia in the children. This association could be partly be explained on the basis that all family members shared the same socioeconomic environment [23].

Our results also showed that prevalence of anaemia among children whose mothers attended antenatal care at least four times was significantly lower and were less likely of becoming anaemic. This is understandable because access to quality healthcare services affords women the opportunity to take advantage of essential preventive measures (nutrition education, promotion of use of insecticide treated nets) thereby ensuring that children are healthy. The finding supports previous studies that have reported the association between infrequent prenatal consultations and the presence of anaemia [46,48,49].

From previous studies, we know that infectious diseases were a common cause of anemia, and anemic children were more susceptive to infectious diseases [50]. Consistent with these findings, having fever in the past two weeks prior to the study was found to be positively related to anemia in this study [51]. Sick children are known to have poor appetite and hence a low dietary intake [52].

In the present study, the children from households without a toilet facility were more likely to have anaemia than their counterparts with at least a pit latrine. Other studies conducted among preschool children elsewhere have highlighted the potential effect of poor water, sanitation and hygiene (WASH) conditions of the physical environment on nutritional status and anaemia [51,53]. Poor sanitation conditions are most likely to be prevalent in environments where there are no decent toilet facilities and such conditions are associated with greater numbers of infectious and parasitic diseases, which in turn can bring about diminishing haemoglobin levels, through poor intake of food and absorption of available nutrients including iron and zinc. Low intake of iron-rich foods and diminished nutrient absorption caused by changes to the gastrointestinal epithelium of infected children can significantly contribute towards anaemia.

The set of predictors of anaemia identified in this study could explain only 17% of the variation in anaemia in the population, which suggest many more factors other than those measured in this study are responsible for anaemia.

## Conclusion and recommendations

The prevalence of anaemia was of public health significance according to the WHO cut-off points. In bivariate analyses, minimum dietary diversity was positively associated with mean Hb concentrations among children 6–23 months but it was negatively associated with Hb concentrations among children 48–59 months. Similarly, minimum dietary diversity was weakly associated with reduced prevalence of anaemia only among children 6–23 months although this relationship disappeared in the multiple regression model.

The current study showed that factors other than poor dietary diversity predicted anaemia among children aged 6–59 months in Ghana. Poverty, child having reported to have high temperature (fever) in the past two weeks, age of the child, continued breastfeeding, anaemic mother, birth interval ≤ 24 months, home delivery and having no access to toilet facility were positively associated with anemia. An integrated approach is required to address the multiple risks. Measures to control anaemia should therefore include alleviating general poverty, empowering and educating women, improving personal and environmental hygiene, improving access to antenatal care services and controlling frequent births.

## Limitations of the study

Although the study used nationally representative data, there were still some limitations that need to be noted and taken into consideration in the interpretation of the of the study results. The cross-sectional nature of this study does not ensure causality between independent variables and anaemia. Anaemia status from the study group was determined on the basis of haemoglobin concentrations, which are not the best indicator compared to serum ferritin. Then, also, recall of food intake in the past 24 hours cannot best reflect usual food intake.

It was also not possible to measure all the potential causes of anaemia (e.g. infections, genetic characteristics and micronutrient interactions may impair normal haemoglobin synthesis). This was manifested in the fact that all of the risk factors studied could only explained 17% of the variation in the outcome. There is therefore the need to conduct a longitudinal study, which may also allow for measurement of a longer exposure to dietary intake.
